# A novel gene, *Le-Dd10,* is involved in fruiting body formation of *Lentinula edodes*

**DOI:** 10.1007/s00203-022-03206-z

**Published:** 2022-09-05

**Authors:** Akihiro Kishikawa, Satoshi Hamada, Ichiro Kamei, Yosuke Fujimoto, Kazuhiro Miyazaki, Motonobu Yoshida

**Affiliations:** 1 Department of Agricultural Science, Kinki University, Nakamachi 3327-204, Nara, 631-8505 Japan; 2 Kyushu Research Center, Forest Products Research Institute, Kurokami 4-11-16, Kumamoto, 860-0862 Japan; 3 Osaka University of Comprehensive Children Education, Yusato 6-4-26, Higashisumiyoshi-ku, Osaka, 546-0013 Japan

**Keywords:** *Dictyostelium discoideum*, Fruiting body, Shiitake mushroom, Transcription factor, Transformation

## Abstract

The cDNA library prepared from *Lentinula edodes*, Hokken 600 (H600), primordia was screened using cDNA expressed specifically in *Dictyostelium discoideum* prestalk as a probe. Twenty-one clones, *Le-Dd1* ~ *21,* were isolated from the *L. edodes* primordia cDNA library. Functional analysis of each gene was carried out by transformation into protoplast cells from *L. edodes* Mori 252 (M252) mycelia with the overexpression vector pLG-RasF1 of each gene because M252 protoplast cells were transformed with an 11-fold higher efficiency than H600 cells. Transformants with the overexpression vector of *Le-Dd10* formed a fruiting body at almost the same time as H600, a positive control, although M252, a negative control, did not form a fruiting body under culture conditions. This suggested that *Le-Dd10* is involved in the formation of fruiting bodies. Single-strand conformation polymorphism analysis revealed that *Le-Dd10* is located on No. 4 linkage group of *L. edodes*. The properties of Le-Dd10 products were investigated by Western blotting analysis using polyclonal antibodies against GST:Le-Dd10 fusion proteins. As a result, 56-kDa, 27-kDa, and 14-kDa protein bands appeared in primordial and fruiting body stages, although the expected molecular weight of the Le-Dd10 product was 50 kDa.

## Introduction

One of the most common edible basidiomycetes among Japanese cultivated mushrooms is shiitake mushroom, *Lentinula edodes*. To produce high-quality shiitake mushrooms efficiently, the application of molecular breeding methods by artificial gene introduction is needed, in addition to crossbreeding by hybridization among cultivars as in past breeding. The mechanism of fungi fruiting body formation has been analyzed in many studies (Kües 2000; Palmer and Horton 2006). Several mutants and genes related to fruiting body development have been isolated in *Coprinopsis cinerea* as a model fungus, including fruiting body maturation, *mat*; stipe elongation, *eln*, *eln2*, *eln3*; cap expansion, *exp1*, *ich1*; basidiospore formation, and *bad* (Arima et al. 2004; Srivilai et al. 2006; Muraguchi et al. 2008). Genes expressed specifically in fruiting bodies, *Le.flp1*, *mfbA, mfbc, and recQ,* were isolated in *L. edodes* (Yasuda and Shishido 1999; Miyazaki et al. 2004, 2007; Katsukawa and Shishido 2005), but it is unknown whether these genes are involved in fruiting body formation of *L. edodes*. It is difficult to identify genes involved in fruiting body formation because of the culture conditions of *L. edodes.* For fruiting body formation, a long period of 2–3 months and laboratory culture system are required. Therefore, in this study, we used the cellular slime mold *Dictyostelium discoideum* cDNA as a probe to detect candidate genes involved in *L. edodes* fruiting body formation because *D. discoideum* cells are capable of forming a fruiting body in 24 h. Using knockout by BSR cassette (blasticidin resistance gene) insertion, each knockout mutant of three cDNA clones, namely, *SSJ301 *(Accession No. AJ488770), *SSJ337* (Accession No. AF161253), and *SSK864* (Accession No. AF140044), exhibited aberrant fruiting bodies (Takamoto et al. 2001; Kamei et al., 2003, 2013). These cDNA clones were used as a probe to screen candidate genes involved in fruiting body formation from the *L. edodes* primordial cDNA library. The transformants in which each endogenous gene of *Le-Dd1* ~ *21* of *L. edodes* was overexpressed were prepared and analysis of most phenotypes was attempted. The phenotypes of the transformants were examined and the effects of overexpressed genes on fruiting body formation were investigated. *Le-Dd10* findings will be used to isolate short-term culture varieties of *L. edodes*.

## Materials and methods

### Strains and culture

*Dictyostelium discoideum* wild-type AX2 was cultivated at 22 °C in HL5 medium. The 45 knockout mutants by BSR cassette containing *bsr* (blasticidin resistance gene) (Sutoh 1993) insertion of *D. discoideum* cDNA clones expressed specifically in stalks were prepared and grown in HL medium containing 10 μg/mL of blasticidin S (Funakoshi, Tokyo, Japan) (Sakuragi et al. 2005). These *D. discoideum* cDNA clones were provided from the “*Dictyostelium* cDNA project in Japan, https://nenkin.nbrp.jp/ clone/list” (Morio et al. 1998). Development was started by washing the cells in 17 mM phosphate buffer, pH 6.1. The cells were agitated on a rotary shaker at 150 rpm or spread on agar plates, and incubated at 22 °C as reported previously (Yoshida et al. 1991). *Lentinula edodes* mycelia were grown on MYPG agar (0.25% malt extract, 0.1% yeast extract, 0.1% peptone, 0.5% glucose, and 0.5% low melting temperature agar) plates at 25 °C. Mycelia agar plate discs were used for inoculation into sawdust pots and culture was continued at 22 °C for 90–150 days. For single-strand conformation polymorphism (SSCP) analysis, parental dikaryon, MCR14, was generated from the monokaryon D703PP-9 (mating type: *A*_*1*_*B*_*1*_) obtained from D703, a New Zealand wild-type strain and the monokaryon G408PP-4 (*A*_*2*_*B*_*2*_) obtained from G408, a Japanese wild-type strain. The 23 tetrads were analyzed in the present study (Miyazaki and Neda 2004; Miyazaki et al. 2014).

### Preparation of a knockout construct and transformation of *Dictyostelium discoideum*

The knockout vector by BSR insertion into the 45 *D. discoideum* cDNA clones was constructed (Sakuragi et al. 2005). A BSR cassette was inserted into the restriction enzyme site of each cDNA in pBluescript KS (-), followed by PCR-mediated amplification using r*Taq* DNA polymerase, T3, and T7 primers (Merck, Darmstadt, Germany). PCR products were purified using QIAGEN-tip 20 (QIAGEN, Duesseldorf, Germany). The knockout construct was transformed into AX2 cells by electroporation, and transformants were independently isolated and analyzed by Southern and Northern blotting. The knockout mutants of three clones, *SSJ301*, *SSJ337*, and *SSK864*, formed aberrant fruiting bodies. Information on each knockout mutant has already been published (*SSK864*, Takamoto et al. 2001; *SSJ301*, Kamei et al. 2003; *SSJ337*, Kamei et al. 2013).

### Preparation of *Lentinula edodes* protoplast

Protoplast cells were prepared from *L. edodes* Mori 252 (M252) mycelia. Protoplast cells from M252 were transformed with 11-fold higher efficiency than those from *L. edodes* Hokken 600 (H600) (Fujimoto et al. 2004). M252 mycelia was inoculated into MYPG agar and cultured in the dark at 25 °C, and used as an inoculum. They were inoculated into 30 mL of twofold concentrated MYPG liquid medium and cultured with shaking at 96 rpm at 25 °C for 3 days. The cultured mycelia were transferred to a homogenizer cup and crushed in a homogenizer at 9,000 rpm for 5 min on ice. Twenty mL of crushed mycelial fragments was filtered through a 100-μm cell strainer (BD Biosciences, MA, USA), added to 20 mL of MYPG liquid medium, and statically cultured for 4 days. They were washed with 10 mL of SM buffer (50 mM succinate, pH 5.6, and 0.6 M mannitol). To lyse mycelial cell walls for protoplast isolation, 1 g wet weight of mycelia was suspended in 10 mL of 2.5% cellulase Onozuka RS (Yakult Honsha, Tokyo, Japan) and 1.0% Lysing Enzyme (Merck)/SM buffer in a centrifuge tube. The tube was installed horizontally, and enzyme treatment was carried out for 4 h with shaking at 60 rpm at 28 °C.

### Cloning of *Lentinula edodes* genes

For the *L. edodes* primordia cDNA library, total RNA of *L. edodes* H600 primordia was extracted using TRIzol^®^Reagent (Invitrogen, Carlsbad, CA, USA). Two hundred μL of TRIzol was added to 0.1 g of primordia and pulverized in a mortar. Poly (A)^+^ RNA was purified from total RNA using a poly (A)^+^ RNA purification kit (GM Healthcare, Buckinghamshire, UK) according to the manufacturer's specifications. The cDNA was made from 5 µg of poly (A)^+^ RNA using a cDNA synthesis kit (GM Healthcare). It was blunted, ligated to *Eco*RI adaptors, phosphorylated, and size-fractionated by column chromatography. Fractions containing cDNA of longer than 500 base pairs were collected, precipitated by ethanol, and ligated into λZAP II arms (Agilent Technologies, CA, USA). The ligated DNA was packaged using a GIGA Pack III Gold packaging kit (Stratagene, La Jolla, CA). The cDNA library was screened using ^32^P-labeled *SSJ301*, *SSJ337*, and *SSK864* cDNA, respectively, as a probe. Random labeling was performed with a Radprime DNA labeling system (Invitrogen) and 20 µCi [α-^32^P]dCTP (GM Healthcare) according to the manufacturer’s protocol. Hybridization was carried out at 42 °C overnight in 6 × SSPE containing 0.05% nonfat dried milk and 50% formamide. Filters were first washed in 2 × SSC, 0.1% SDS at room temperature, and then in 1 × SSC, 0.1% SDS at 68 °C. Positive clones were sequenced on an ABI Prism^®^3100-Avant Genetic Analyzer (Applied Biosystems, Foster City, CA, USA) using BigDye^®^Terminator v3.1 Cycle Sequencing Kit (Applied Biosystems). The sequences were then compared with those registered in the GenBank™ data bank. Open reading frames were predicted using GENETXY-MAC 8.0. Homology searches of nucleotide and deduced amino acid sequences were carried out using BLASTX (DDBJ; http://www.ddbj.nig.ac.jp/welcome-j.html) and Simple Modular Architecture Research Tool (SMART; http://smart.embl-heidelberg.de/smart/ change_mode.pl). *Le-Dd1*–*Le-Dd7* clones were isolated by *SSJ301* with 38.1–55.1% homology, *Le-Dd8*–*Le-Dd12* clones by *SSJ337* with 41.8–45.3% homology, and *Le-Dd13*–*Le-Dd21* clones by *SSK864* with 42.1–50.6% homology.

### Transformation of *Lentinula edodes*

The *L. edodes* expression vector pLG-RasF1 with *hph* (hygromycin resistance gene) was constructed using the promoter of *the Le. Ras* gene (Hori et al. 1991) and constitutively expressed in *L. edodes* cells (Fujimoto et al. 2004). Each gene, *Le-Dd5, Le-Dd6, Le-Dd7, Le-Dd9, Le-Dd10, Le-Dd11, Le-Dd12, Le-Dd13, Le-Dd14,* and *Le-Dd18,* was inserted into pLG-RasF1 digested with *Sma*I and dephosphorylated. Each cDNA expression vector was constructed (Hamada et al. 2008). For transformation of *L. edodes* M252 protoplasts, the REMI method (Sato et al. 1998) was used in which gene transfer into protoplasts was carried out by osmotic action. STC buffer (10 mM Tris–HCl, pH 7.5, 10 mM CaCl_2_, and 1.2 M sorbitol) was added to the protoplast suspension to be 0.5 to 1.0 × 10^7^/240 μL. Ten μL of STC buffer containing 10 μg of the cDNA expression vector and 10 units of *Sph*I (or *Dra*I or *Bsp*T104I) was added to the tube. It was gently stirred and incubated on ice for 20 min. Next, 62.5 μL of a PEG solution of 60% PEG 4000 (Nacalai tesque, Kyoto, Japan) was added and incubated on ice for 20 min. Thereafter, 3.125 mL of the PEG solution was further added, and the mixture was kept at 25 °C for 20 min. Next, 10 mL of STC buffer was added. After centrifugation at 2,500 rpm at 4 °C for 10 min, the precipitate suspended in 4 mL of MS medium (2.0% malt extract and 0.6 M sucrose) was transferred to a plastic dish (BD Biosciences). The transformed protoplasts were cultured in an incubator at 25 °C in the dark for 3 days. They were then applied to minimal agar medium (Kües 2000), pH 4.5, 2.0 g of agar/100 mL, including 0.2% Sanpearl CP (Nippon Paper Chemicals, Tokyo, Japan) and 5 μg/mL of hygromycin B (Nacalai tesque). They were cultured in an incubator at 25 °C in the dark for 4 days. Thereafter, only the culture solution was removed, and 9 mL of MYPG agar medium (0.5% low melting temperature agarose) including 20 μg/mL of hygromycin B was layered. Colonies that appeared after stratification were cultured in an incubator at 25 °C in the dark. The most marginal part of the mycelial tuft was punched with a cork borer. It was inoculated into 30 mL of MYPG medium supplemented with 0.2% Sanpearl CP and cultured in an incubator for 14 days.

### Culture of *L. edodes* on block medium (*Castanopsis* sawdust medium)

*Castanopsis* sawdust and wheat bran were sterilized in an autoclave at 121 °C for 60 min and then mixed with the composition rate of 36% sawdust, 4% bran, and 60% DW. The mixture (90 g) was placed in a cultivation pot sterilized at 121 °C for 10 min, and was again sterilized at 121 °C for 60 min in an autoclave. The transformed mycelia that were cultured in MYPG agar medium for 14 days were punched out with a cork borer. Nine disks were inoculated into a cultivation pot. There were three cultivation periods for *L. edodes* mycelia: 90, 120, and 150 days, cultivation was performed with three cultivation pots for each category under 90% humidity and light irradiation at 22 °C, and the growth rate of mycelia was measured. When the period of each culture was reached, sterilized water was added to the cultivation pot in the clean bench until the medium was immersed with sterilized water. Low-temperature treatment was performed at 4 °C for 17 h, and sterile water in the cultivation pot was removed in the clean bench. Culture was continued under 90% humidity and light irradiation at 15 °C to promote fruiting body formation. The average days required for fruiting body formation and the number of cultivation pots forming a fruiting body after the low-temperature treatment were measured with three cultivation pots for each category.

### Southern blotting and Northern blotting analyses using *L. edodes* cDNA

A cDNA probe was labeled with digoxigenin (DIG) by the random hexamer procedure using a DIG DNA labeling kit (Roche Diagnostics, Mannheim, Germany). Genomic DNA was extracted from *L. edodes* mycelia with 2.0% Cetyl trimethyl ammonium bromide (CTAB) in 100 mM Tris–HCl, pH 8.0, 1.4 M NaCl, 20 mM EDTA, and 0.2% β-mercaptoethanol, and total RNA was extracted with ISOGEN (Nippon Gene, Toyama, Japan) from *L. edodes* mycelia or transformants with the RNAi vector for *Le-Dd10*. Southern blotting and Northern blotting analyses were conducted using each cDNA as a probe. Twenty μg of genomic DNA digested with an appropriate restriction enzyme and ten μg of total RNA were separated on an agarose gel. After agarose gel electrophoresis, agarose gels were transferred onto Hybond™ N^+^nylon membrane (GM Healthcare) with VacuGeneXL (GM Healthcare). Hybridization was carried out at 42 °C for Southern blotting analysis and at 50 °C for Northern blotting analysis in DIG easy hyb (Roche Diagnostics).

### Construction of RNAi for *Le-Dd10*

The RNAi vector for *Le-Dd10* was constructed with a 146-nucleotide sequence comprising the 40-bp short homologous hairpin dsRNA sequence, a flanking sequence (6 nucleotide), and a 60-nucleotide spacer sequence, as described in a previous paper (Nakade et al. 2011). To construct the *Le-Dd10* homologous inverted repeat sequence expression vector (pivrLe-Dd10), two 146-base oligonucleotide sequences LeDd10ivrF and LeDd10ivrR (Table 1) were synthesized by Eurofins genomics (Tokyo, Japan). These contained a 40-bp homologous inverted repeat sequence from *Le-Dd10* exon connected to a 60-nucleotide linked loop sequence from intron 2 of *Lcc1.* These oligonucleotides were heated to 105 °C for 10 min and allowed to spontaneously anneal for 30 min while cooling to room temperature. After annealing, the oligonucleotides were blunted and the insert was ligated into the dephosphorylated *Sma*I-digested pLG-RasF1 vector. The cultivation period of each transformant mycelium for RNAi-1, RNAi-2, RNAi-18, and RNAi-19, including Le-Dd10-3, Le-Dd10-5, M252, and H600 as a control, was divided into 90, 120, and 150 days. The average number of days required for fruiting body formation was measured with three cultivation pots for each category.

**Table 1:** List of primers used in this study

**Primer for GST fusion protein**
**GSTLeDd10F** 5′-GGAGGATCCATGTCGAGTTTTGC-3′
**GSTLeDd10R** 5′-CGGGAATTCATTTCGTCGATAG-3′
**Primer for RNAi construction**
**LeDd10ivrF**
5′-TCGACAACTTCGCGAGCGAAAACTGCAGGCGGAGCTTAGGCGCCA
**ATTGTATGTCACTCCAGCACGATTTGACAGCGGTAGCTTCATCGTCCCTATTCCTAGCAT**
TGGCGCCTAAGCTCCGCCTGCAGTTTTCGCTCGCGAAGTTG-3′
**LeDd10ivrR**
5′-TCGACAACTTCGCGAGCGAAAACTGCAGGCGGAGCTTAGGCGCCA **ATGCTAGGAATAGGGACGATGAAGCTACCGCTGTCAAATCGTGCTGGAGTGACATACAAT**
TGGCGCCTAAGCTCCGCCTGCAGTTTTCGCTCGCGAAGTTG-3′

Bold letters indicate the intron2 region of *Lcc1*

### Construction of the GST:Le-Dd10 fusion protein expression vector and preparation of a polyclonal antibody against Le-Dd10 products

To prepare GST:Le-Dd10, *Le-Dd10* cDNA fragments were prepared with the primers GSTLeDd10F and GSTLeDd10R (Table 1), digested with *Bam*HI and *Eco*RI, and inserted into the *Bam*HI and *Eco*RI site of pGEX-2 T, a GST fusion protein expression vector (GM Healthcare). After transforming into *Escherichia coli* BL21-Gold (DE3) cells (Agilent Technologies, CA, USA), transformants were incubated with 0.5 mM IPTG. GST:Le-Dd10 fusion proteins were adequately expressed at 37 °C for 16 h. The GST:Le-Dd10 was cut out from a 10% gel of sodium dodecyl sulfate-polyacrylamide gel electrophoresis (SDS-PAGE). A 50-kDa target band was formed from the 26 kDa of GST and 24 kDa of Le-Dd10 protein. Gel slices from cells incubated at 37 °C for 16 h were stirred in 50 mM Tris–HCl buffer (pH 8.1) containing 0.1% SDS, 5% β -mercaptoethanol, 1 mM EDTA, and 0.2 mM phenylmethylsulfonyl fluoride at room temperature overnight. They were then suspended in PBS and subcutaneously and intraperitoneally injected into male rabbits to prepare the polyclonal antibody as previously described (Yoshida 1987). Four injections were performed at intervals of 10 days and rabbits were bled to death 1 week after the last injection. As control serum, sera were withdrawn from rabbits before the first injection.

### Western blotting analysis of the Le-Dd10 product

*Lentinula edodes* mycelia, primordia, fruiting body stipe, and pileus were separately suspended in PBS, and sonicated at level 4 for 30 s three times at intervals of 30 s with a Branson sonifier 250 (Branson Ultrasonics, CT, USA). They were mixed with the same volume of 2X SDS-PAGE sample buffer and boiled in a water bath for 5 min. They were then applied to a 12.5% SDS-PAGE gel following a standard method (Laemmli 1970) with a slight modification. After electrophoresis, SDS-PAGE gels were transferred to a PVDF membrane (Atto, Tokyo, Japan). The membrane was incubated with a polyclonal antibody against GST:Le-Dd10 fusion proteins diluted 1:1000 as the primary antibody, and then with peroxidase-conjugated goat anti-rabbit IgG (Organon Teknika Corp., NC, USA) diluted 1:500 as the secondary antibody. Western blotting analysis using the polyclonal antibody against GST:Le-Dd10 fusion proteins revealed a 50-kDa major single band of fusion proteins. No signals were observed using control serum.

### PCR amplification for SSCP analysis

PCR amplification was used to detect polymorphisms by designing specific primers and amplifying fragments for SSCP analysis. Each reaction mixture consisted of 20 mM Tris–HCl, pH 8.5, 50 mM KCl, 2.5 mM MgCl_2_, 0.16 mM each dNTP, 0.08 μM each primer, 5 ng of genomic DNA, and 0.25 units of Platinum *Taq* DNA polymerase (Invitrogen). The total volume of each reaction mixture was 12.5 μL, which was overlaid with mineral oil. The thermal cycling conditions were as follows: 30 cycles at 95 °C for 30 s, 55 °C for 90 s, and 72 °C for 120 s, followed by 10-min incubation at 72 °C and subsequent cooling to 4 °C (PE480 thermal cycler; Applied Biosystems, Foster, CA, USA).

### Single-strand conformation polymorphism (SSCP) analysis

Biotin-labeled PCR products were diluted 50- to 100-fold in 1 X TBE buffer (89 mM Tris–HCl, pH 8.0, 89 mM boric acid, and 2 mM EDTA), 6% (w/v) sucrose, and 0.33% tartrazine. Double-stranded DNA in a diluted solution was denatured and maintained at 96 °C for 5 min, and then the mixture was cooled on ice. The mixture was subsequently loaded on a 15 × 40-cm vertical 5% Hydro-Link Long acrylamide gel (BMA, Maine, USA) in 1 X TBE buffer and subjected to electrophoresis at 14 °C for 90 min at 30 W. DNA samples were transferred to a nylon transfer membrane (MSI, MA, USA) and visualized using a Phototope-Star Detection Kit (New England Biolabs, MA, USA).

## Results and discussion

### Isolation of *Lentinula edodes* cDNA clones

**Fig. 1:**
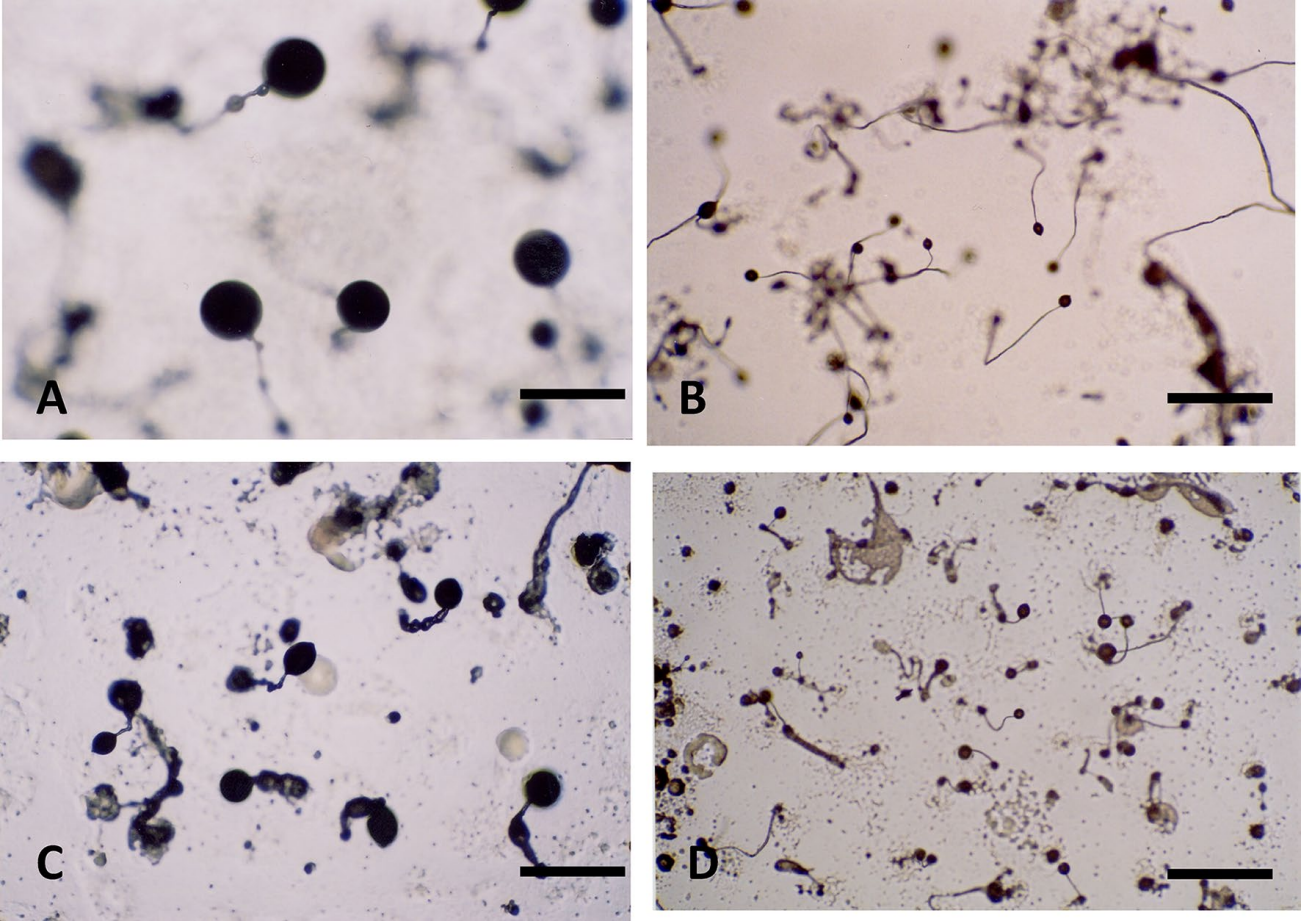
Knockout mutants of cDNA clones, SSJ301, SSJ337, and SSK864 exhibited aberrant fruiting bodies. **A** AX2 Control; **B** SSJ301; **C** SSJ337; **D** SSK864 SSJ301, a fruiting body with a tiny sorus; SSJ337, a fruiting body with an abnormal stalk; SSK864, a tiny fruiting body Scale bars are 50 μm

We aimed to isolate genes involved in the fruiting body formation of *Lentinula edodes*. However, there were some difficulties in this project: the long cultural period of *L. edodes* and confirmation of fruiting body formation in laboratory culture systems. Therefore, at the first step, we attempted to use *Dictyostelium discoideum* cDNA, which are able to form fruiting bodies in 24 h, as probes to isolate the target genes involved in fruiting body formation from *L. edodes* cells. Knockout constructs for the 45 *D. discoideum* cDNA clones, whose transcripts were specifically expressed in *D. discoideum* prestalks, were prepared as described in Materials and Methods. Among the 45 knockout mutants, mutants for three cDNA clones, namely, *SSJ301* (Accession No. AJ488770) encoding oxysterol-binding protein, *SSJ337* (Accession No. AF161253) encoding protein phosphatase 4, and *SSK864* (Accession No. AF140044) encoding calmodulin-binding protein, exhibited aberrant fruiting bodies; SSJ301, a fruiting body with a tiny sorus; SSJ337, a fruiting body with an abnormal stalk; SSK864, a tiny fruiting body (Fig. 1; Takamoto et al. 2001; Kamei et al 2003, 2013). The *L. edodes* primordia cDNA library was screened using ^32^P-labeled *SSJ301, SSJ337, and SSK864* cDNA as a probe. As a result, 21 cDNA clones, *Le-Dd1*–*21,* were isolated, and the properties of each cDNA clone are summarized in Table 2.

**Table 2:** cDNA clones isolated from the *L. edodes* primordial cDNA library

Clone name	Related protein^a^	Homology^a^	Size^b^ (kb)	Existence of 5′ end^c^	Position of domain^d^
*Le-Dd1*	*Cryptococcus neoformans* putative uncharacterized protein	129/335 (38%)	1.2	No	
*Le-Dd2*	*Candida albicans* BNI4	24/82 (29%)	0.9	No	
*Le-Dd3*	*Cryptococcus neoformans* DNA-binding protein Ncp 1	80/314 (25%)	1.4	No	
*Le-Dd4*	*Dictyostelium discoideum* ORF	23/57 (40%)	3.2	No	
*Le-Dd5*	*Cryptococcus neoformans* putative uncharacterized protein	50/126 (39%)	1.2	Yes	Transmembrane 5-27aa
*Le-Dd6*	*Shewanella baltica* glycosyltransferase, group1	24/93 (25%)	1.4	Yes	None
*Le-Dd7*	*Cryptococcus neoformans* NADH-ubiquinone oxidoreductase 51 kDa subunit	392/482 (81%)	1.5	Yes	None
*Le-Dd8*	hydrophobin 1	127/127 (100%)	0.6	Yes	HYDRO 53-122aa
*Le-Dd9*	*Ustilago maydis* 40S ribosomal protein S12	108/146 (73%)	0.6	Yes	None
*Le-Dd10*	*Schizosaccharomyces pombe* non-specific DNA-binding protein Spt2	64/254 (25%)	1.7	Yes	SPT2 324-440aa
*Le-Dd11*	*Schizosaccharomyces pombe* pre-mRNA-splicing factor srp2	77/167 (46%)	1.1	Yes	RRM 4–69, 98–166
*Le-Dd12*	*Cryptococcus neoformans* putative uncharacterized protein	138/432 (31%)	1.6	Yes	None
*Le-Dd13*	hydrophobin 1	127/127 (100%)	0.6	Yes	HYDRO 53-122aa
*Le-Dd14*	*Neosartorya fischeri* high-affinity methionine permease	151/322 (46%)	2.0	Yes	Transmembrane 30–52,73–95,110–132,144–166 aa
*Le-Dd15*	*Cryptococcus neoformans* aspartokinase	110/266 (41%)	2.0	No	
*Le-Dd16*	*Coprinus cinereus* subtilisin-like serine protease	231/483 (47%)	1.7	No	
*Le-Dd17*	*Cryptosporidium hominis* ATP-dependent RNA helicase	41/62 (66%)	0.9	No	
*Le-Dd18*	*Neosartorya fischeri* yipee zinc-binding protein	66/106 (62%)	1.2	Yes	None
*Le-Dd19*	*Cryptococcus neoformans* heat shock protein	212/286 (74%)	2.0	No	
*Le-Dd20*	*Salinibacter ruber* putative uncharacterized protein	15/35 (42%)	0.6	No	
*Le-Dd21*	*Cryptococcus neoformans* cyclophilin	51/115 (44%)	0.8	No	RRM 10–89

^a^Homology search was performed using BLASTX program on DDBJ server

^b^cDNA size was calculated from nucleotide sequences

^c^Existence of 5’ end was predicted from nucleotide sequences

^d^Domain in full-length cDNA genes was predicted with SMART program

### *Lentinula edodes* genes involved in fruiting body formation

**Fig. 2:**
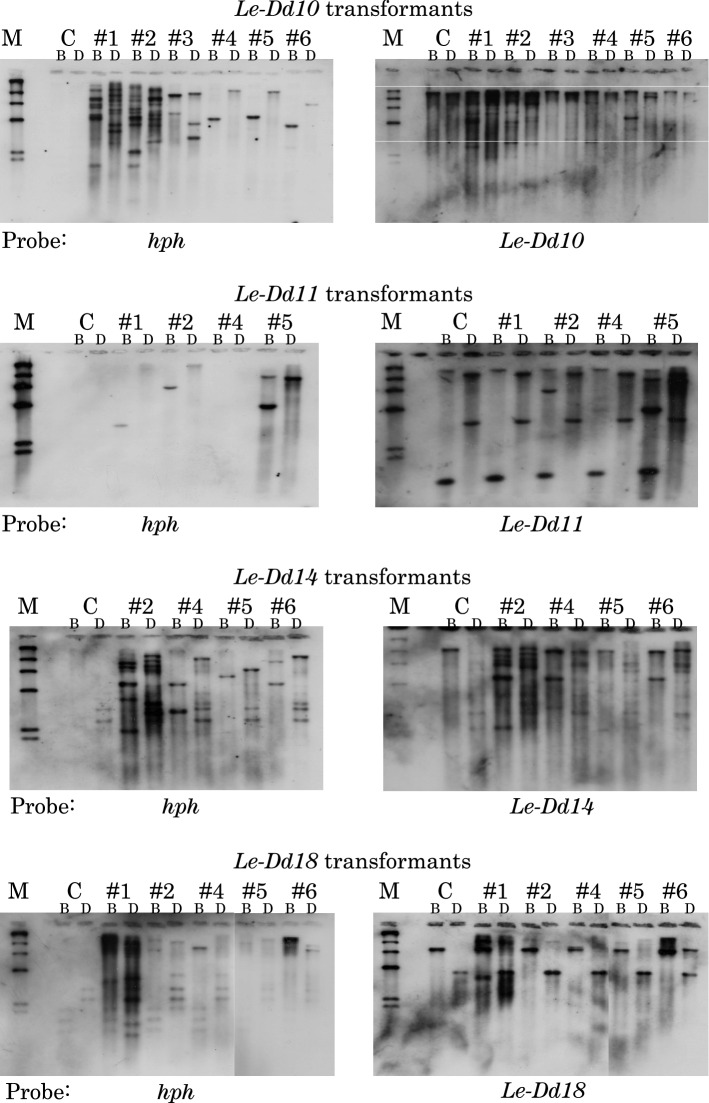
Southern blot analysis of transformants with *Le-Dd10*, *Le-Dd11*, *Le-Dd14*, or *Le-Dd18*. Southern blotting was performed using *Le-Dd10*, *Le-Dd11*, *Le-Dd14*, or *Le-Dd18* cDNA and *hph* (hygromycin resistance gene) as a probe. The symbol # indicates a clone of each transformant. The genomic DNA of *L. edodes* mycelia was digested with *Bam*H I or *Dra* I. M, λDNA digested with *Hin*d III; C, Mori 252; B, *Bam*H I; D, *Dra* I

**Fig. 3:**
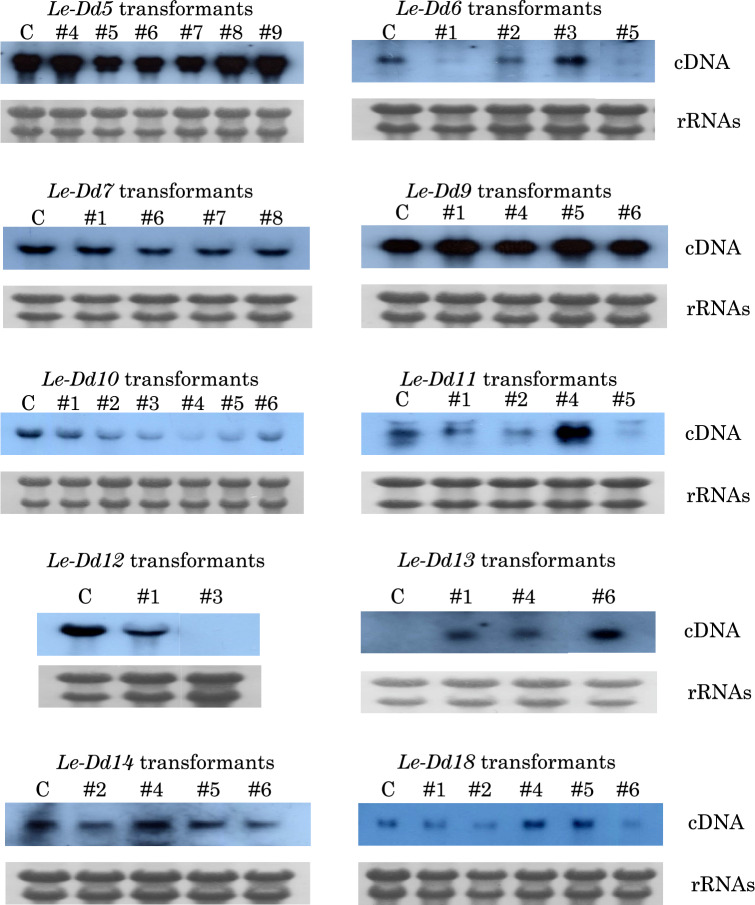
Northern blot analysis of pLG-RasF1/Le-Dd5, Le-Dd6, Le-Dd7, Le-Dd9, Le-Dd10, Le-Dd11, Le-Dd12, Le-Dd13, Le-Dd14, and Le-Dd18. Northern blotting was performed using insert cDNA as a probe. The symbol # indicates a clone of each transformant. Total RNA was isolated from transformant mycelia cultured at 25 °C for 2 weeks C, Mori 252

Among 21 *L. edodes* cDNAs, the 10 cDNAs, *Le-Dd5*, *Le-Dd6*, *Le-Dd7*, *Le-Dd9*, *Le-Dd10*, *Le-Dd11*, *Le-Dd12*, *Le-Dd13*, *Le-Dd14*, and *Le-Dd18*, which had the 5’-terminal end, except for *Le-Dd8*, were inserted into the pLG-RasF1 vector with *hph* (hygromycin resistance gene) as described in the Materials and Methods. *Le-Dd8* and *Le-Dd13* cDNAs were a known gene, hydrophobin 1. The expression vector of each cDNA, pLG-RasF1/ Le-Dd5, pLG-RasF1/Le-Dd6, pLG-RasF1/Le-Dd7, pLG-RasF1/ Le-Dd9, pLG-RasF1/Le-Dd10, pLG-RasF1/Le-Dd11, pLG-RasF1/Le-Dd12, pLG-RasF1/Le-Dd13, pLG-RasF1/Le-Dd14, and pLG-RasF1/Le-Dd18, was constructed (Hamada et al. 2008). Transformation of *L. edodes* protoplasts was performed by the REMI method. Transformant colonies were screened, isolated, and grown on MYPG agar medium. Furthermore, they were cultured on block medium (*Castanopsis* sawdust medium) to reach a fruiting body stage. The cultivation period of *L. edodes* mycelia was divided into 90, 120, and 150 days and cultivation was carried out as described in Materials and Methods. The growth rate of mycelia was measured. As a result, no differences in the growth rate were observed among each transformant, including Hokken 600 (H600) and Mori 252 (M252). Representative clone results (Le-Dd10, Le-Dd11, Le-Dd14, and Le-Dd18) of Southern blotting analysis of each transformant mycelium are shown in Fig. 2. New bands not found in M252 as a control were detected in four clones, showing successful transformation. In the other clones (Le-Dd5, Le-Dd6, Le-Dd7, Le-Dd9, Le-Dd12, and Le-Dd13), new bands were also identified by Southern blotting analysis. The results of Northern blotting analysis for each transformant mycelium are shown in Fig. 3. Different transcripts were found among each clone. In transcripts of each transformant clone of Le-Dd5, Le-Dd7, Le-Dd9, and Le-Dd14, no significant differences were observed among transformant clones, including M252 as a control. However, high and low transcripts were detected in each transformant clone of Le-Dd6, Le-Dd10, Le-Dd11, Le-Dd12, Le-Dd13, and Le-Dd18. These results exclude the possibility of contamination with another clone or strain, including H600 or M252. If contamination occurs in transformants, different types of transcripts do not appear among each clone.

When each culture period reached 90, 120, or 150 days, culture was continued under 90% humidity and light irradiation at 15 °C to promote fruiting body formation after treatment as described in Materials and Methods. The number of days required for fruiting body formation after low-temperature treatment was measured (Table 3). The period to reach fruiting body formation for the *Le-Dd10* cDNA overexpression clone (clones 10#3 ~ 10#6) was shorter than that for the other overexpression clones (Table 3), irrespective of the mRNA level of *Le-Dd10* in mycelia (Fig. 3). Regarding the *Le-Dd18* cDNA overexpression clone, the period to reach fruiting body formation differed between clone 18#4 and clone 18#6, showing different insertion states in genomic DNA between the two clones (Fig. 2). The overexpression vector including each cDNA as an insert was introduced into the protoplast cells from M252, which was unable to form fruiting bodies in fungal bed cultivation (block medium of *Castanopsis* sawdust), and was only able to form fruiting bodies in timber cultivation. On the other hand, H600 formed fruiting bodies in fungal bed cultivation. Therefore, the period to reach fruiting body formation for each overexpression clone of *Le-Dd10* cDNA was shorter than that for the other overexpression clones or almost the same time as H600, the positive control, suggesting that Le-Dd10 products have properties that stimulate fruiting body formation.

**Table 3:** The number of days required for fruiting body formation

Clone	Period					
	90 days culture		120 days culture		150 days culture	
5#7	–	–	–	–	ND	ND
5#9	–	–	–	–	ND	ND
6#3	–	–	52^a^	2^b^	40	2
6#5	–	–	–	–	43	2
7#6	–	–	19	2	14	3
9#4	–	–	–	–	–	–
9#5	–	–	72	2	ND	ND
10#3	75	2	20	2	ND	ND
10#4	56	1	15	3	15	3
10#5	63	1	22	2	14	3
10#6	54	1	15	3	15	3
11#4	–	–	62	1	30	2
12#3	–	–	–	–	34	3
13#6	–	–	35	2	ND	ND
14#4	–	–	–	–	ND	ND
14#6	–	–	–	–	ND	ND
18#4	–	–	–	–	36	3
18#6	50	2	16	3	13	3
H600	72	1	45	2	38	2
M252	–	–	–	–	–	–
RasF1	–	–	–	–	–	–

Clone name, Clone 7 of *Le-Dd5* was indicated as 5#7

RasF1, transformant with pLG-RasF1; H600 (Hokken 600), positive control; M252 (Mori 252), negative control

The symbol – indicates that fruiting bodies were not formed, *ND* indicates that nothing was done

^a^Average days required for fruiting body formation

^b^The number of the pots in which fruiting bodies were formed

**Fig. 4:**
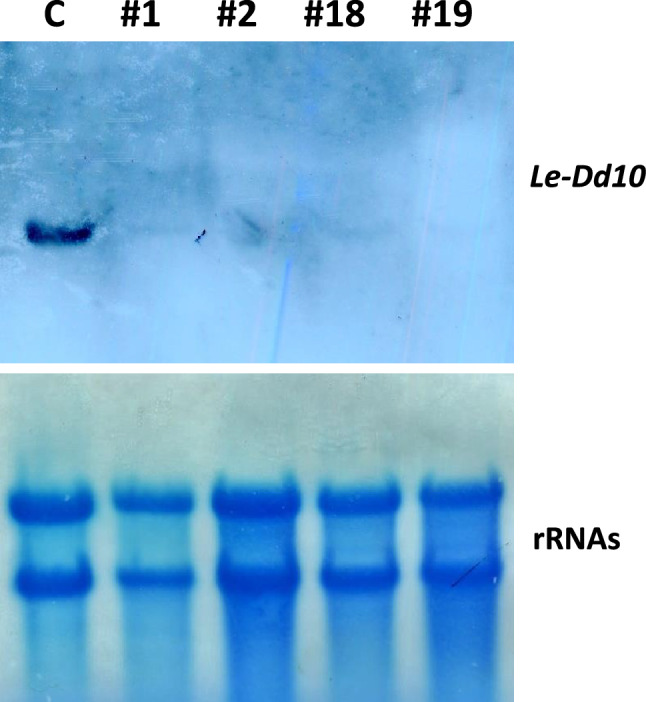
Northern blot analysis of transformants with RNAi vector for *Le-Dd10*. Northern blotting was performed using *Le-Dd10* cDNA as a probe. The symbol # indicates a clone of each transformant. Total RNA was isolated from each mycelium transformed with the RNAi vector cultured at 25 °C for 2 weeks C, Mori 252

Next, we prepared the RNAi vector for *Le-Dd10* with the intron 2 region of *Lcc1* as described in Materials and Methods. The results of Northern blotting analysis of transformants with the RNAi vector for *Le-Dd10* are shown in Fig. 4. In these transformants, no significant differences were observed in the growth rate among each transformant mycelium, including H600 and M252, and the property to stimulate fruiting body formation in Le-Dd10 was not detected in each transformant for RNAi-1, RNAi-2, RNAi-18, or RNAi-19 under the culture conditions described in the Materials and Methods.

### Mapping of *Le-Dd10* on the linkage of *Lentinula edodes*

**Fig. 5:**
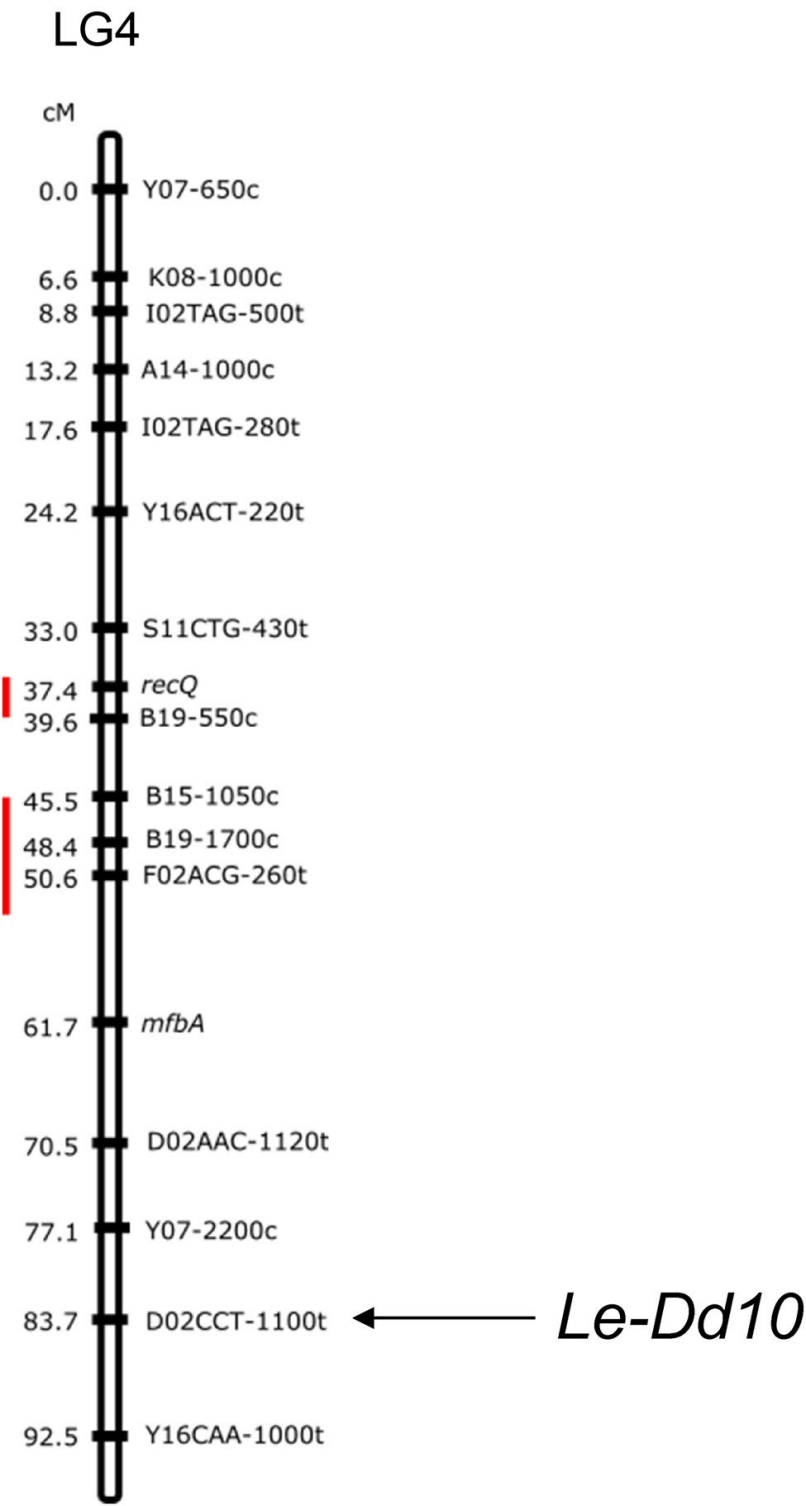
Mapping of *Le-Dd10* on the linkage of *Lentinula edodes.* Mapping of *Le-Dd10* was carried out by SSCP analysis. An arrow shows the position of *Le-Dd10*

Mapping of the *Le-Dd10* gene was performed by tetrad analysis, PCR amplification, and single-strand conformation polymorphism (SSCP) analysis, as reported previously (Miyazaki and Neda 2004; Miyazaki et al. 2014). PCR amplification and SSCP analysis revealed that the *Le-Dd10* gene was mapped to linkage group LG4 among 11 linkage groups (Fig. 5). Both genes of *recQ* (Katsukawa and Shishido 2005) and *mfbA* (Yasuda and Shishido 1999) were located on LG4. They were transcribed in fruiting bodies during *L. edodes* development.

### Analysis of Le-Dd10 products by Western blotting analysis

**Fig. 6:**
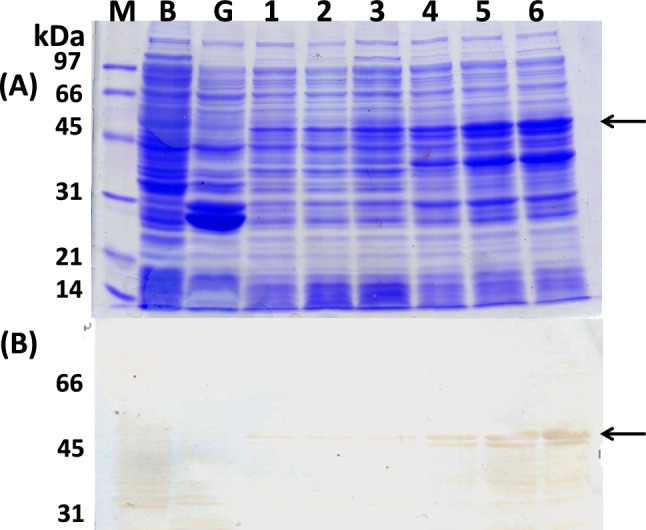
SDS-PAGE of GST:Le-Dd10 fusion proteins. The transformants with the GST:Le-Dd10 fusion expression vector were incubated with 0.5 mM IPTG at 25 °C or 37 °C for 5, 10, or 16 h. They were applied on a 10% gel and the gel was stained with Coomassie brilliant blue (**A**). The PVDF membrane for Western blotting analysis was incubated with anti-GST antibody diluted 1:1000 as a primary antibody and then incubated with peroxidase-conjugated goat anti-rabbit IgG diluted 1:500 as a secondary antibody (**B**). M, molecular weight marker; B, *Escherichia coli* BL21- Gold (DE3) without expression vector; G with GST expression vector; 1–6 with GST:Le-Dd10 fusion expression; 1–3 at 25 °C, 4–6 at 37 °C; 1, 4 for 5 h; 2, 5 for 10 h; 3, 6 for 16 h. Arrows show the position of a fusion protein band

**Fig. 7:**
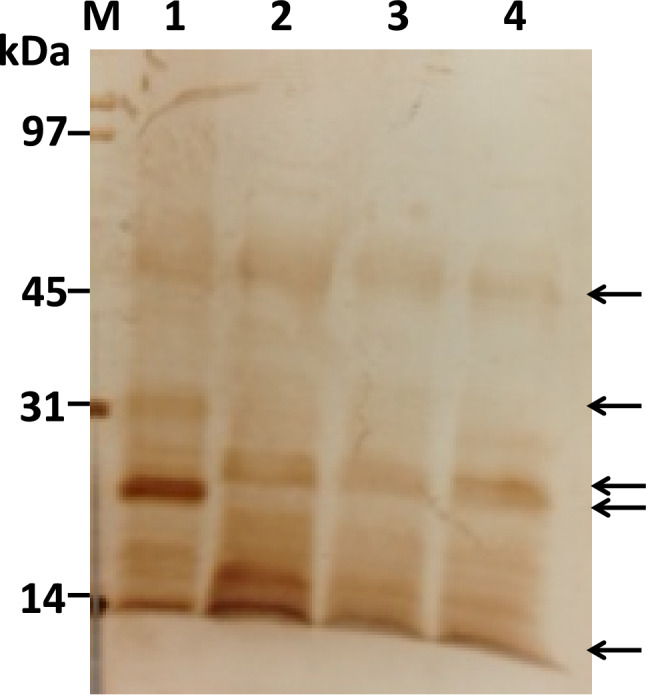
Western blot analysis of Hokken 600. Each sample was prepared as described in Materials and Methods. Arrows show the positions of 56-kDa, 31-kDa, 27-kDa, 24-kDa, and 14-kDa bands. M, SDS-PAGE marker; 1, mycelium; 2, primordium; 3, fruiting body stipe; 4, fruiting body pileus

To prepare GST:Le-Dd10, a GST-Le-Dd10 fusion protein expression vector was constructed and GST:Le-Dd10 was isolated as described in Materials and Methods. After transforming into *Escherichia coli* BL21-Gold (DE3) cells, transformants were incubated with 0.5 mM IPTG at 25 °C or 37 °C for 5, 10, 16 h. GST:Le-Dd10 fusion proteins were adequately expressed at 37 °C for 16 h. The GST:Le-Dd10 was cut out from a 10% gel of sodium dodecyl sulfate-polyacrylamide gel electrophoresis (SDS-PAGE). A 50-kDa target band was formed from the 26 kDa of GST and 24 kDa of Le-Dd10 protein (Fig. 6). Most GST:Le-Dd10 became insoluble in culture conditions of *E. coli* BL21-Gold cells, even at a lower temperature of 18 °C. Therefore, solubilization of GST:Le-Dd10 was attempted using 0.1% TritonX-100, Tween20, CHAPS, and other detergents. Only SDS of higher than 0.1% was effective for the solubilization of GST:Le-Dd10, but GST:Le-Dd10 solubilized with SDS did not bind the GST affinity column. The GST:Le-Dd10 fusion proteins as an antigen for producing the polyclonal antibody were prepared by cutting and extracting the 50-kDa target bands from *E. coli* BL21-Gold cells incubated at 37 °C for 16 h. Western blotting analysis using the polyclonal antibody against GST:Le-Dd10 revealed that the molecular weight band of 56 kDa was observed in primordial and fruiting body stages of H600, and that major bands of 27 kDa and 14 kDa were observed in primordial and fruiting body stages of H600. On the other hand, the major bands of 56, 31, 24, and 14 kDa were observed in the mycelial stage of H600 (Fig. 7), with the *Le-Dd10* transcript being detected in M252 mycelia (Fig. 3). The function of the Le-Dd10 protein expressed in the mycelial stage may be different from that in the primordial and fruiting body stages, suggesting that the Le-Dd10 product is a bifunctional protein.

The expected molecular weight of the Le-Dd10 product is 50 kDa based on the total amino acid sequence. However, the band position on SDS-PAGE was 56 kDa. The Le-Dd10 product had three predicted sites for N-linked sugar chains, and many predicted sites for O-linked sugar chains and phosphorylation. The differences between the expected molecular weight and that observed on SDS-PAGE may be due to the modification of sugar chains or other modifications, including phosphorylation. The Le-Dd10 protein has a Spt2 domain with a helix-turn-helix, similar to a DNA-binding protein (Hershkovits et al. 2006; Nourani et al. 2006). The Spt2 domain is located at the C-terminal site of the Le-Dd10 protein. Among the three predicted sites for N-linked sugar chains, one is near the Spt2 domain and two are within the domain. More than two-thirds of the total predicted sites for O-linked sugar chains and phosphorylation exist at the C-terminal site of the Le-Dd10 product. Thus, the Le-Dd10 protein may function as a transcription factor in fruiting body formation, suggesting that the DNA-binding activity of Le-Dd10 protein is regulated by the Spt2 domain, and reciprocal modifications of sugar and phosphorylation (Hart et al. 2007).

The relationship among the 56-kDa, 27-kDa, and 14-kDa proteins in primordial and fruiting body stages is unknown. However, for the Le-Dd10 protein, the change from 56 to 14 kDa may be important for the stimulation of fruiting body formation. Using different varieties of *L. edodes*, XR-1, KS11, KA1001, H600, H607, and H715, Western blotting analysis was carried out. Among them, the varieties with shorter culture periods to form fruiting bodies had strong expression of the 14-kDa protein in the primordial or fruiting body stage. Previous findings demonstrated that the modification of a transcription factor changed its activity (Klenova et al. 1997; Tootle and Rebay 2005). Therefore, it is conceivable that the 14-kDa protein may be produced from a 56-kDa protein through protease digestion.
